# Seeing Your Stories: Visualization for Narrative Medicine

**DOI:** 10.34133/hds.0103

**Published:** 2024-01-23

**Authors:** Hua Ma, Xiaoru Yuan, Xu Sun, Glyn Lawson, Qingfeng Wang

**Affiliations:** ^1^ Faculty of Science and Engineering, University of Nottingham, Ningbo 315100, China.; ^2^ Digital Art Department, Art & Design Technology Institute, Suzhou 215104, China.; ^3^ National Key Laboratory of General Artificial Intelligence and School of Intelligence Science and Technology, Peking University, Beijing 100871, China.; ^4^ Health Data Visualization and Visual Analytics Research Center, National Institute of Health Data Science at PKU, Beijing 100191, China.; ^5^ Nottingham Ningbo China Beacons of Excellence Research and Innovation Institute, University of Nottingham Ningbo China, Ningbo 315100, China.; ^6^ Human Factors Research Group, Faculty of Engineering, University of Nottingham, Nottingham NG7 2RD, UK.; ^7^ Nottingham University Business School China, University of Nottingham, Ningbo 315100, China.

## Abstract

**Importance:** Narrative medicine (NM), in which patient stories play a crucial role in their diagnosis and treatment, can potentially support a more holistic approach to patient care than traditional scientific ones. However, there are some challenges in the implementation of narrative medicine, for example, differences in understanding illnesses between physicians and patients and physicians’ increased workloads and overloaded schedules. This paper first presents a review to explore previous visualization research for narrative medicine to bridge the gap between visualization researchers and narrative medicine experts and explore further visualization opportunities. **Highlights:** The review is conducted from 2 perspectives: (a) the contexts and domains in which visualization has been explored for narrative medicine and (b) the forms and solutions applied in these studies. Four applied domains are defined, including understanding patients from narrative records, medical communication, medical conversation training in education, and psychotherapy and emotional wellness enhancement. **Conclusions:** A future work framework illustrates some opportunities for future research, including groups of specific directions and future points for the 4 domains and 3 technological exploration opportunities (combination of narrative and medical data visualization, task-audience-based visual storytelling, and user-centered interactive visualization). Specifically, 3 directions of future work in medical communication (asynchronous online physician-patient communication, synchronous face-to-face medical conversation, and medical knowledge dissemination) were concluded.

## Introduction

The respected physician Francis W. Peabody criticized the contemporary medical practice in 1926, arguing that it was only concerned with applying scientific concepts and methods of medicine. According to Peabody, effective treatment also requires a more holistic understanding, and physicians should have a broader view of their profession. Hence, listening to patients and establishing a patient–doctor relationship to learn about their personalities, backgrounds, likes, and dislikes is crucial for effective treatment [1]. However, it was not until the mid-1980s that the connection between narrative and healthcare practice became a concern [2–4]. Around this time, the terms “Narrative-Based Primary Care”, “Narrative-Based Medicine”, and “Narrative Medicine” appeared [5–7]. In 2001, Rita Charon, an internist and literary scholar at Columbia University, described “Narrative Medicine” as “medicine practised with narrative competence” as well as “a model for humane and effective medical practice” [7]. Here, the narrative competence required refers to acknowledging, absorbing, interpreting, and acting on the stories of patients [7]. By reading, writing, telling, and receiving stories in their medical practice, physicians can reach and join their patients in their illnesses and empathize with their situations [8,9]. The goal of narrative medicine in clinical practice is to develop a shared personalized treatment plan, to make patients more satisfied with their care providers, and to help them achieve a better life [10,11].

However, several barriers have always affected the practice of narrative medicine. For example, Charon [8] described the differences between patients’ and physicians’ understanding and experiences of illness. Physicians consider illness from a narrow, biological angle and regard death as a technical failure. In contrast, patients think of illness as a whole life and see death as unthinkable. Moreover, when understanding the causes of symptoms, patients and professionals often have a profound disagreement, inevitably preventing effective care. Hence, physicians’ awareness of the holistic view of patients as people and their ability to build good patient–doctor relationships and empathize with their patients’ disease experiences is a crucial element of clinical practice. However, according to Engel [12], more specialized physician training (in particular in organs and organ systems) leads to a narrower focus and more possibilities for medical students to lose a complete view of patients. Meanwhile, a lack of time in clinical encounters impedes medical practitioners from developing empathy with patients and reflecting on their own practices [12]. Hence, increased workloads and overloaded schedules are generally severe obstacles in medical practice [13,14].

In current data-driven medical practice, research into medical visualization is an area of current research interest [15]. Medical visualization technology focuses mainly on enabling medical professionals to access medical evidence in real time and to aid stakeholders in understanding clinical data results. Medical visualization visualizes the complex results of diverse health data analyses, including multiomics biological data, medical images, electronic health records (EHRs), physiologic data, and other behavioral or environmental data [15]. This kind of support saves time and improves the ability of physicians to access and assimilate a large amount of healthcare data [16,17]. However, aside from statistical-evidence-based scientific analysis and diagnosis, the practices of narrative medicine need another perspective of anecdotal-evidence-based support of visualization technologies, substantially enhancing health communication with patients [13,18]. Anecdotal evidence, as opposed to statistical evidence, includes narrative elements that can form connections with patients’ characters and situations. Furthermore, the communication of anecdotal evidence does relate to the experiences and emotions, not only statistical evidence [18,19].

To date, despite the interest in medical visualization in the academic literature, few studies have reviewed and analyzed visualizations in narrative medicine practice. For example, Gillmann et al. [20] highlighted 10 open challenges around scientific medical data visualization and Preim and Lawonn [21] reviewed visual analytics techniques employed in public health. Several surveys focused on specific visualization techniques in medical practices: Zhou et al. [22] reviewed 3-dimensional (3D) visualization techniques for medical imaging data and Preim and Meuschke [23] emphasized medical animations. Moreover, Meuschke et al. [24] explored narrative visualization technologies applied to visualize data-driven stories about diseases. Nevertheless, there is no survey article on visualization in narrative medicine. More visualization solutions for narrative medicine practice should be explored.

Hence, this article presents a review of the state-of-the-art of visualization research for narrative medicine. The exploration was conducted from 2 perspectives: (a) the contexts and domains in which visualization has been explored for narrative medicine and (b) the forms and solutions applied in these studies. In the following, we will provide an overview of the exploration regarding existing visualization works and illustrate a future work framework to discuss the potential issues and future directions while highlighting the trends for future research.

## Materials and Methods

In order to explore the existing studies covering narrative medicine and information visualization, we collected studies from their titles, abstracts and keywords with the search terms “narrative”, “medicine”, or its synonyms “illness”, “clinical”, “diagnose” or “healthcare”, and “visualisation” or “visualization” or “storytelling”. Here, “storytelling” is included in the search terms as a synonym of “visualisation”. Storytelling, mainly “serious storytelling”, is applied in narrative medicine to incorporate patients’ experiences and whole life stories into the treatment plans [25]. Meanwhile, storytelling in visualization, e.g., narrative visualization, was demonstrated to aid narrative communication in data-rich domains more effectively and understandable [26]. Therefore, some research works used “storytelling” visualization technologies and methods.

**Fig. 1.**
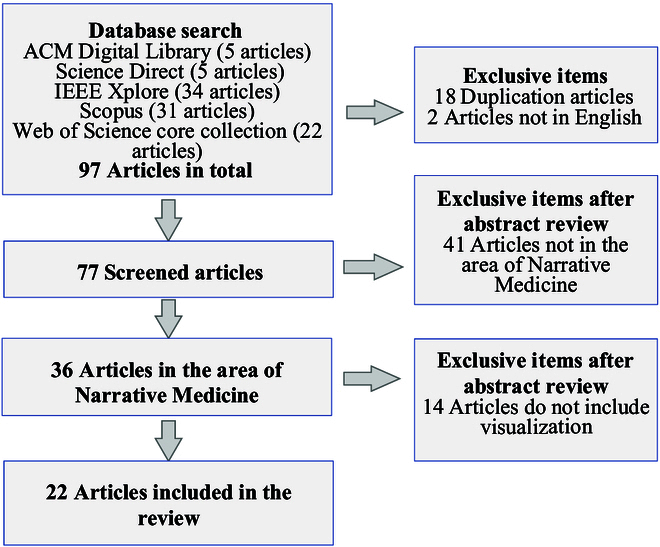
The results of article search and selection process.

We reviewed visualization research works for narrative medicine published since 2001 when Charon first defined narrative medicine. The works were collected from 5 databases, including IEEE Xplore, ACM Digital Library, Scopus, Science Direct, and the Web of Science core collection. Figure 1 illustrates the search results and the exclusion selection in a flow chart. Overall, we collected 97 studies with the previously described search strategies. We excluded the 18 duplicate articles and the 2 that are not in the English language. There were 77 articles remaining. After the abstract review for these articles, we excluded the 41 articles not included in the area of narrative medicine; 36 articles remained. Based on these articles, after excluding the 14 articles without the work of visualization, 22 articles were obtained. In our review study about the range of visualization works, we excluded those that only depended on pictures, photos, and paintings that are not data/info-driven visualizations and are neither created by visualization techniques.

## Visualization in Support of 4 Domains in Narrative Medicine

Following the review of the 22 identified papers, the 4 applied domains or contexts of narrative medicine emerged, as shown in Table 1. These are (a) understanding patients from narrative records, (b) medical communication, (c) medical conversation training in education, and (d) psychotherapy and emotional wellness enhancement. The findings from each domain are presented in Visualization for understanding patients from narrative records, Visualization for medical communication, Visualization for medical conversation training in education, and Visualization for psychotherapy and emotional wellness enhancement below.

**Table 1.** Studies by domains and years

Domain /Year	(1) Understanding patients from narrative records			(2) Medical communication					(3) Medical conversation training in education	(4) Psychotherapy and emotional wellness enhancement
	Medical history overview enhancement	Disease-symptom causal relations exploration	New information discovery	Online asynchronous medical communication	Patient-generated data recording for medical encounters	Health risk communication	Medical knowledge dissemination	Connecting people with diseases		
2005										(22) Coyle et al. [27]
2008									(20) Koyama et al. [28]	
2009	(9) Zhu et al.[29]									
2010	(8) Gschwandtner et al. [30]									
2011	(7) Nair et al.[31]									
2012			(6) Farri et al. [32]							
2013										(21) Choudhury et al. [33]
2014		(5) Park and Choi [34]							(19) Babaian [35]	
2016	(4) Jensen and Bossen [36]					(14) Ottley et al. [37]				
2017						(13) Hakone et al. [38]				
2018		(3) Shah et al. [39]		(11) Rossano and Roselli [40]	(12) Lakshmi et al. [41]					
2019	(2) Sultanum et al.[42]									
2020				(10) So et al.[43]			(18) Botsis et al. [44]			
2021							(16) Madathil and Greenstein [45]	(17) Junior et al. [46]		
2022	(1) Kenei and Opiyo [47]						(15) Meuschke et al. [48]			
Number of works		9				9			2	2

### Visualization for understanding patients from narrative records

In understanding patients from unstructured or narrative records, for example, EHRs, the studies aimed mainly at physicians’ heavy review work in practice. The visualizations were applied to explore approaches for representing information, relationships between diseases and symptoms, new information in clinical documents, and narrative or medication events to support and improve physicians’ understanding of patient’s medical history in their clinical notes. All studies set their target users as physicians (not patients).

#### Enhancement of medical history overview

**Fig. 2.**
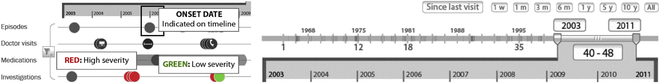
The interactive visualization solution of Nair et al. Left: Different events on a timeline, the event “Onset”, and the severity indications. Right: A zoomable interactive timeline. © [2023] IEEE. Reprinted, with permission, from [31].

Physicians require a quick overview of a patient’s medical history from the EHRs to understand their medical issues and any contextual factors influencing their health (family status, economic situation, mental health, etc.). However, obtaining a rapid and sufficient overview is challenging for physicians because of the large amount of text, their limited available time, and their individual needs [49–52]. To address these problems, Zhu et al. [29] explored how mediation data from a mixed source of coded and narrative text could be displayed. They proposed a temporal visualization system (timeline) to improve medication reconciliation. In their work, the mapping of data resources to event taxonomy and a zoomable bird’s-eye-view time map were designed in the timeline. Nair et al. [31] focused on supporting physicians in understanding a patient’s “story” through an overview of their medical history in the EHR. First, a problem-oriented methodology was used to organize data into meaningful groups. Then, the groups were arranged and displayed together to create the “story”. As shown in Fig. 2, health events (“Onset” and the severity indications, and others) were presented on a zoomable interactive timeline, and detailed interaction provided customized multilevel information exploration.

To explore the factors that affect physicians’ use of overview interfaces, Jensen and Bossen [36] provided a customized and flexible system with different building blocks (reason for contact, diagnosis, previous anesthesia, operations and future appointments, etc.) for physicians in different departments. Quantitative and qualitative analysis found that departments with standard information preferred using the overview interface. However, those departments with long and complex history information did not use the system much. Hence, while the configurable overview interface could support the connection of complex clinical tasks, uncertainty among users discouraged its use.

**Fig. 3.**
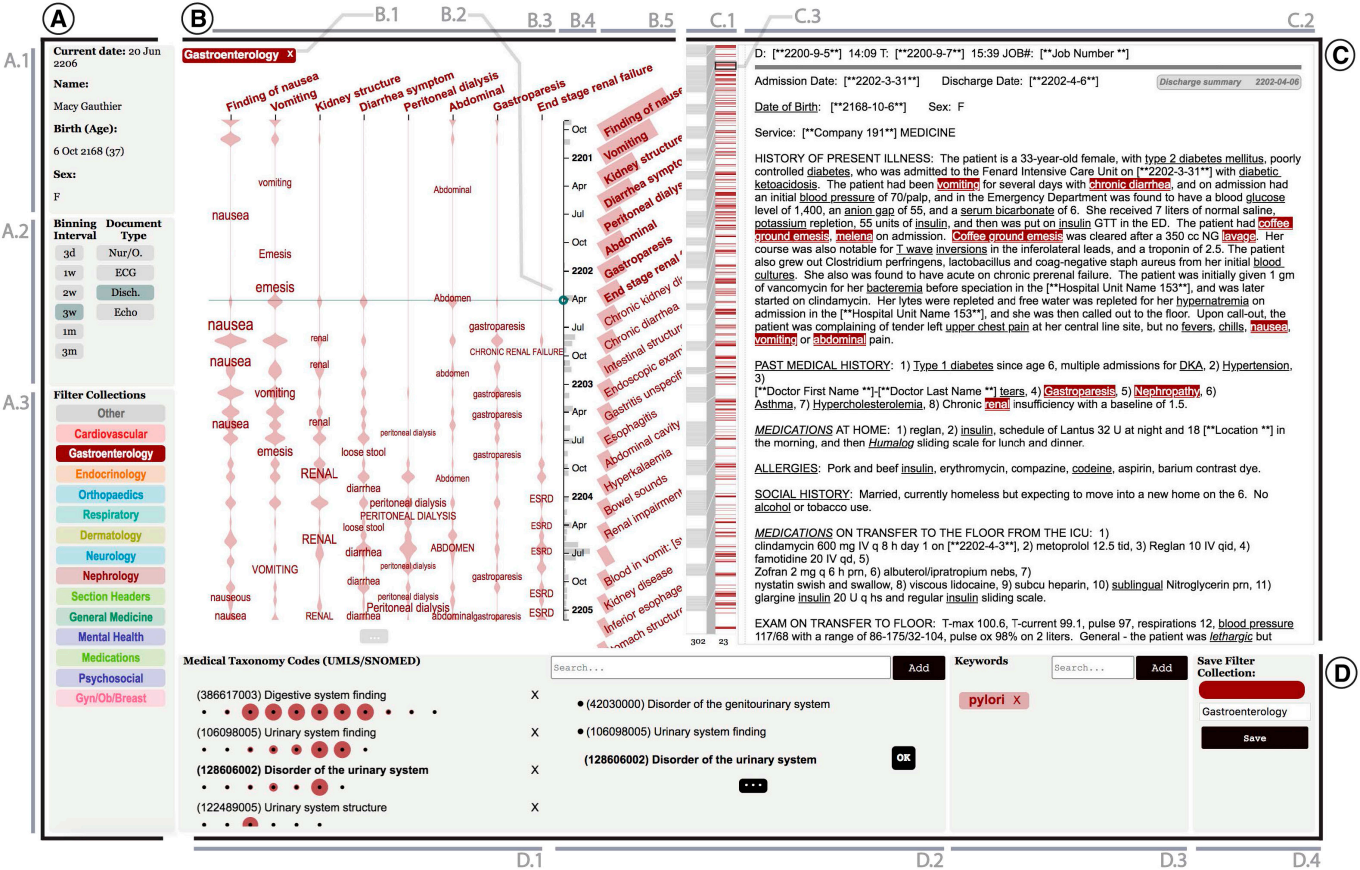
Doccurate by Sultanum et al. 2019. (A) Control panel. (B) Timeline. (C) Text panel. (D) Curation panel. © [2023] IEEE. Reprinted, with permission, from [42].

Sultanum et al. [42] proposed a clinical text visualization approach, Doccurate, to provide an overview of a patient’s medical history in clinical notes. They focused on the need for physicians’ comprehensive review and the limitations of information retrieval algorithms used in visual summaries. Hence, a more physician-centered manner was utilized in their work, and a curation-based interactive visualization approach was explored to support physicians’ different clinical tasks, as illustrated in Fig. 3. Doccurate’s 4 panels (control panel, timeline, text panel, and curation panel) support physicians in defining customizable facets of the medical narrative, correcting errors, and integrating the data visualization tool into daily clinical workflows. Meanwhile, Doccurare is an improvement of automatic medical nature language processing (NLP) [42]. Gschwandtner et al. [30] also provided medical experts with an interactive annotation system and explored visualization features to enhance their navigating and modifying of the annotated semantic information.

Furthermore, Kenei and Opiyo [47] have visually modeled clinical narrative texts in EHRs into different semantic facets (subjective, objective, assessment, and plan). They proposed a visual cluster map layout to assist physicians when reviewing text documents, in which they distinguish different information facets with different colors, text groups, and the presentation of the relationships between the semantic facets. A clinical setup evaluation will be conducted in the future to validate the developed technology.

#### Exploration of disease-symptom causal relations

**Fig. 4.**
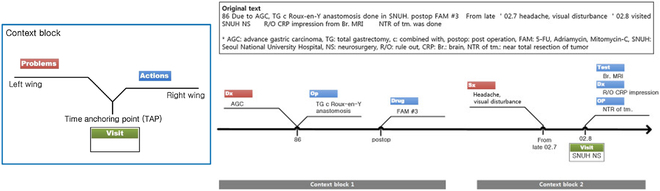
V-model of Park and Choi 2014 [34]. Left: Problem–action causal relations. TAP is temporal information. Right: Part of a clinical text represented in V-model.

Shah et al. [39] present the chronologic relationship between a patient’s diseases and symptoms. They mined the disease concept associations from clinical notes and then visualized the patient’s medical history overview to support their physician’s decision-making. However, the authors report on an insufficient training sample and that the accuracy of this disease concept should be improved and validated further. Meanwhile, the tool cannot deal with 2 different co-occurring concepts. Park and Choi [34] better visualize a clinical narrative into a timeline. They proposed a dynamically scaled V-model timeline to present problem–action causal relations and time anchoring points in a patient’s clinical documents, which appear primarily chronologically. As shown in Fig. 4, the timeline supported temporal reasoning and improved the readability by utilizing uneven granularity expressions (year, hour, or minute levels).

#### Discovery of new information in EHR

Farri et al. [32] focused on visualizing new (nonredundant) information in EHR to synthesize clinical documents. The new information visualization tool includes a demographic panel, clinical document list, document viewer, and review changes tab. They target minimizing erroneous data interpretations, reducing information omission, supporting multitasking, and minimizing interruptions. It has been demonstrated that new information visualization positively influenced information retrieval and increased the ability of clinicians to synthesize information.

### Visualization for medical communication

Moving onto the second domain of medical communication, most of the target users were physicians and patients rather than single physicians. In medical communication, the narrative elements, namely patients’ experiences, feelings, mental states, family histories, and social conditions, should be combined into medical evidence-based data for better health choices [18].

#### Visual tools of on-line asynchronous medical communication

**Fig. 5.**
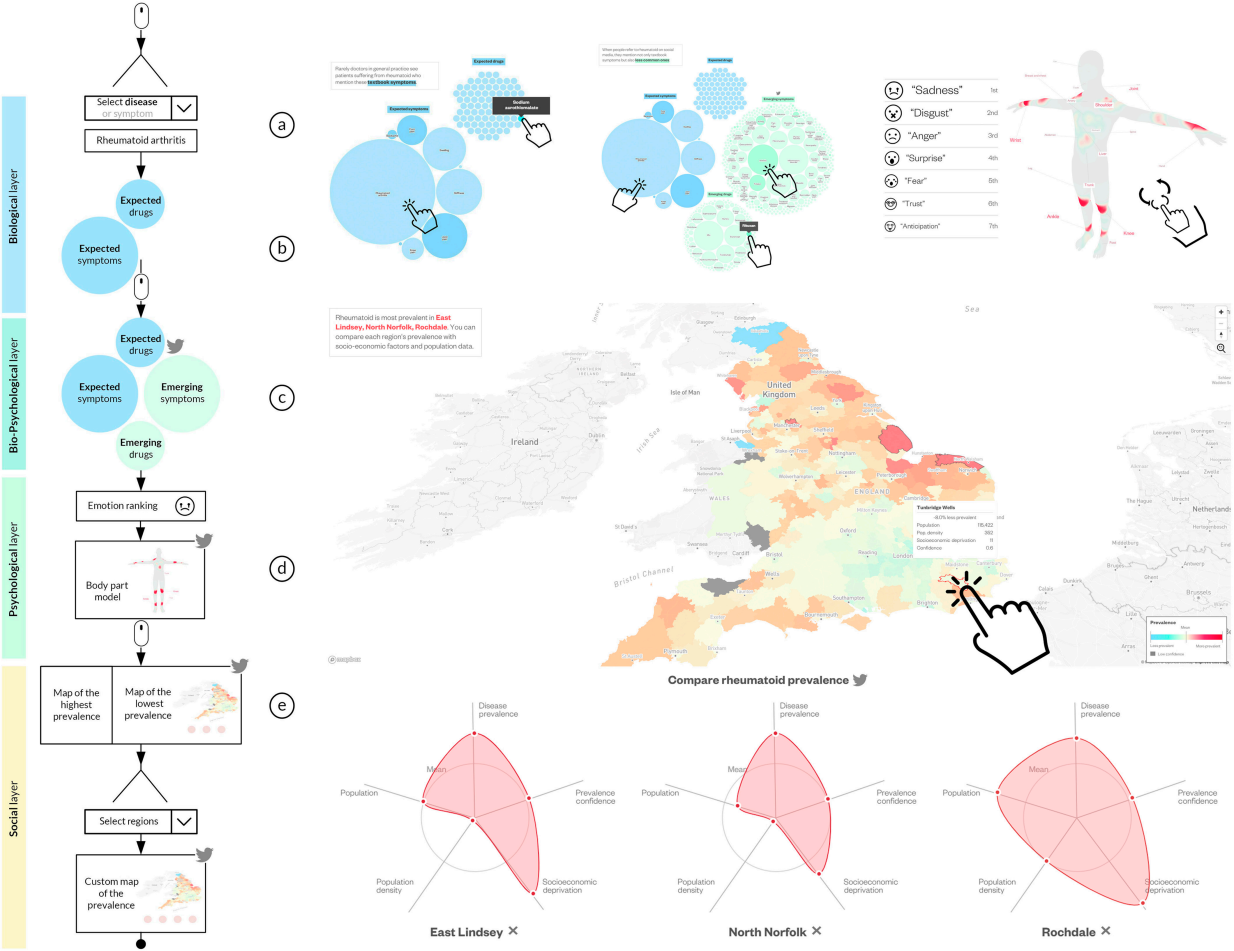
An example of visual storytelling by So, Bogucka et al. 2020. Left: The storyline. (a) Selecting a medical condition. (b) Biological layer. (c and d) Psychological layers. (e) Social layer. Right-top: Biological and phycological parts. The grouped circle charts display expected symptoms (blue bubbles) and emerging symptoms (green bubbles). On the right are the emotions associated with the respective body parts. Right-bottom: Social part. A map of the prevalence of prescriptions. © [2023] IEEE. Reprinted, with permission, from [43].

To support physician-patient communication, So et al. [43] explored visualization approaches for expressing patients’ biological, psychological, and social status information. In their study, social status was people’s medical condition within the areas where patients lived and was related to disease prevalence and census data. From their perspectives, the 3 aspects were equally important in the “bio-psycho-social model” modern healthcare model. They organized all 3 aspects with a narrative visualization structure “martini glass structure” into an interactive dashboard with 3-story slices, as shown in Fig. 5. They suggested that the theoretical implications of adaptive narrative visualization and visualization with human-in-the-loop could be explored in the future. The work context was asynchronous online communication. Another example of work on online and asynchronous communications is from Rossano and Roselli [40]. They targeted young patients with Type 1 diabetes. A game-based learning approach and a digital storytelling solution were developed for engaging and effective communication. Multimedia tools such as graphics, audio, video, and animation were included. With these tools, young patients were supported to acquire knowledge and skills about the disease and to share their emotions and feelings with stakeholders (e.g., physicians, nurses, family, teachers, etc.).

#### Visual recording of patient-generated data for medical encounters

**Fig. 6.**
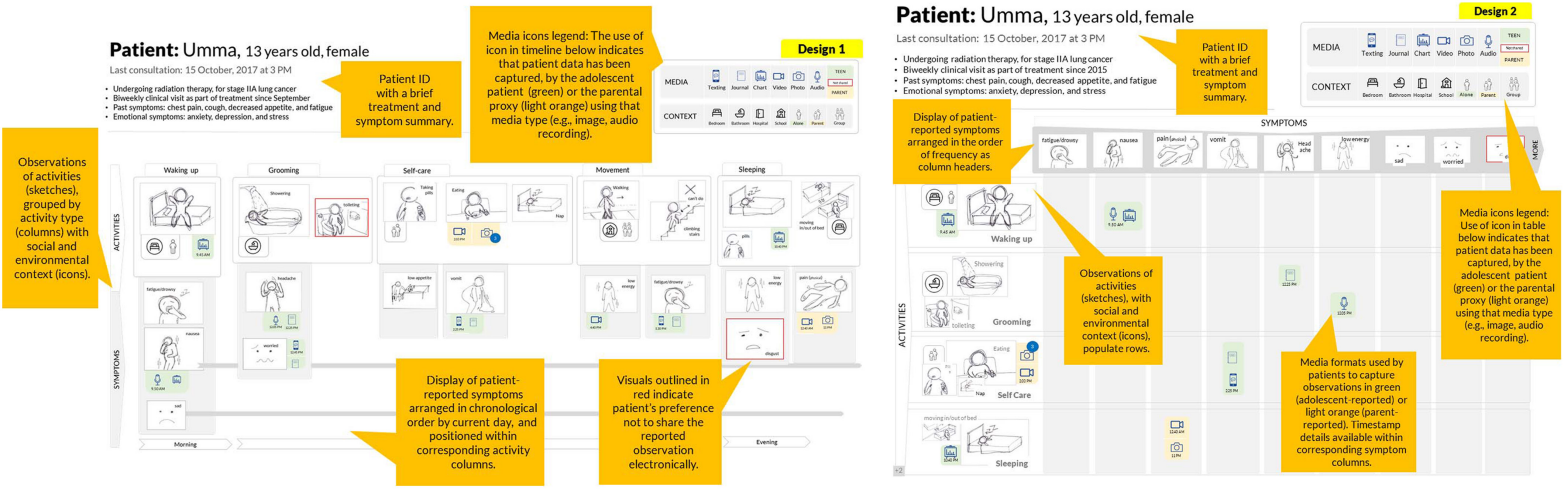
Visual ODLs by Lakshmi, Hong et al. 2018. Left: A daily timeline of patient-generated Visual ODLs. Right: A tabular layout of patient-generated Visual ODLs for reviewing salient data patterns. © [2023] IEEE. Reprinted, with permission, from [41].

Lakshmi et al. [41] not only considered online and asynchronous communication but also began considering scenarios featuring a consultation visit (i.e., a face-to-face encounter). Their study focused on visual reporting and reviewing patient-defined and patient-generated data for medical needs before a consultation or between 2 visits. They suggested that visual narratives of patients’ observations of daily living (Visual ODLs) could contribute to symptom tracking and communication. The 2 views provided by interactive visual ODL dashboards (recording and reviewing) separately supported the activities and symptom recording of young patients and their parent’s and clinical providers’ reviews of their patients’ experiences. See Fig. 6. The Visual ODLs enhanced the effects of patients’ narratives and individual experiences in the whole medical treatment. Their study mentioned scenarios featuring face-to-face conversations; however, this was not their main point.

#### Augmentation of health risk communication

**Fig. 7.**
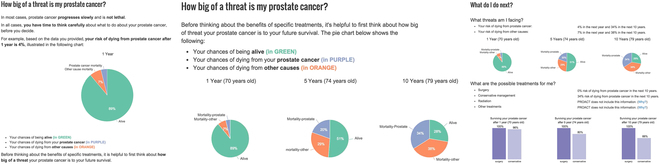
PROACT by Hakone et al. 2017. Left: Presenting 1-year survival rate to “calm down” the patient. Middle: Presenting the 1-,5-, and 10-year mortality rates. Right: Presenting all the information on a summary page to be printed and taken home. © [2023] IEEE. Reprinted, with permission, from [38].

Hakone et al. [38] focused on the health risk communication of severe side effects between patients and physicians. For example, make king shared decisions about treatment plans for prostate cancer, surgery, or conservative treatment. In practice, they are concerned about “the use of quantitative, numeric risk estimates, minimising cognitive burden, defining the reference class, accounting for limitations in numeracy and literacy, conveying uncertainty of risk estimation, and using visualisation for improved comprehension”. Hence, they developed PROACT (PROgnosis Assessment for Conservative Treatment) (see Fig. 7) to support patients’ and doctors’ understanding and communication of cancer risk information. For example, present the 1-year survival rate to “calm down” the patient and the 1-,5-, and 10-year mortality rates to support comparing the survival rates of undergoing surgery or conservative treatment.

**Fig. 8.**
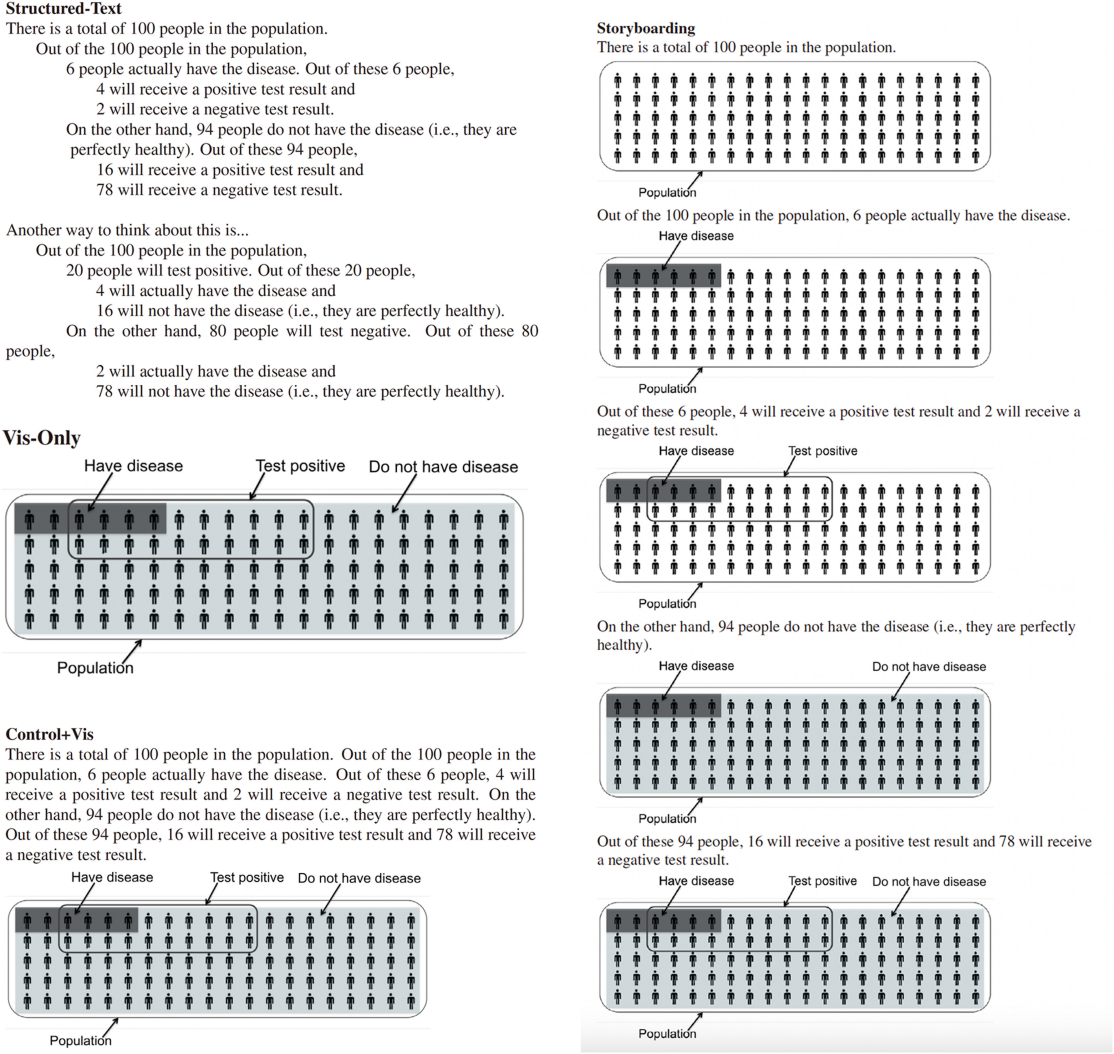
Ottley et al. 2016 - Left-top: Structured text. Left-middle: Vis-Only. Left-bottom: Control text+Vis. Right: Storyboarding. © [2023] IEEE. Reprinted, with permission, from [37].

Similarly, Ottley et al. [37] studied the visualization approaches employed in health risk communication. However, they explored how to increase accuracy for conditional probability estimations by text and visualization design to facilitate Bayesian reasoning. For patients to understand health risk information, conditional probability and Bayesian reasoning were found to be essential. They did experiments for different representations (see Fig. 8), e.g., Control-text, Complete-text, Structured-text, Vis-Only, Control+Vis, and Storyboarding. As a result, they identified that (a) phrasing text could be effective for comprehension, (b) human’s spatial abilities could influence the performance of reasoning tasks, (c) text and visualization combined could produce interference and impede users’ understanding, and (d) storyboarding may create incongruences with the mental model.

#### Complementing medical knowledge dissemination

**Fig. 9.**
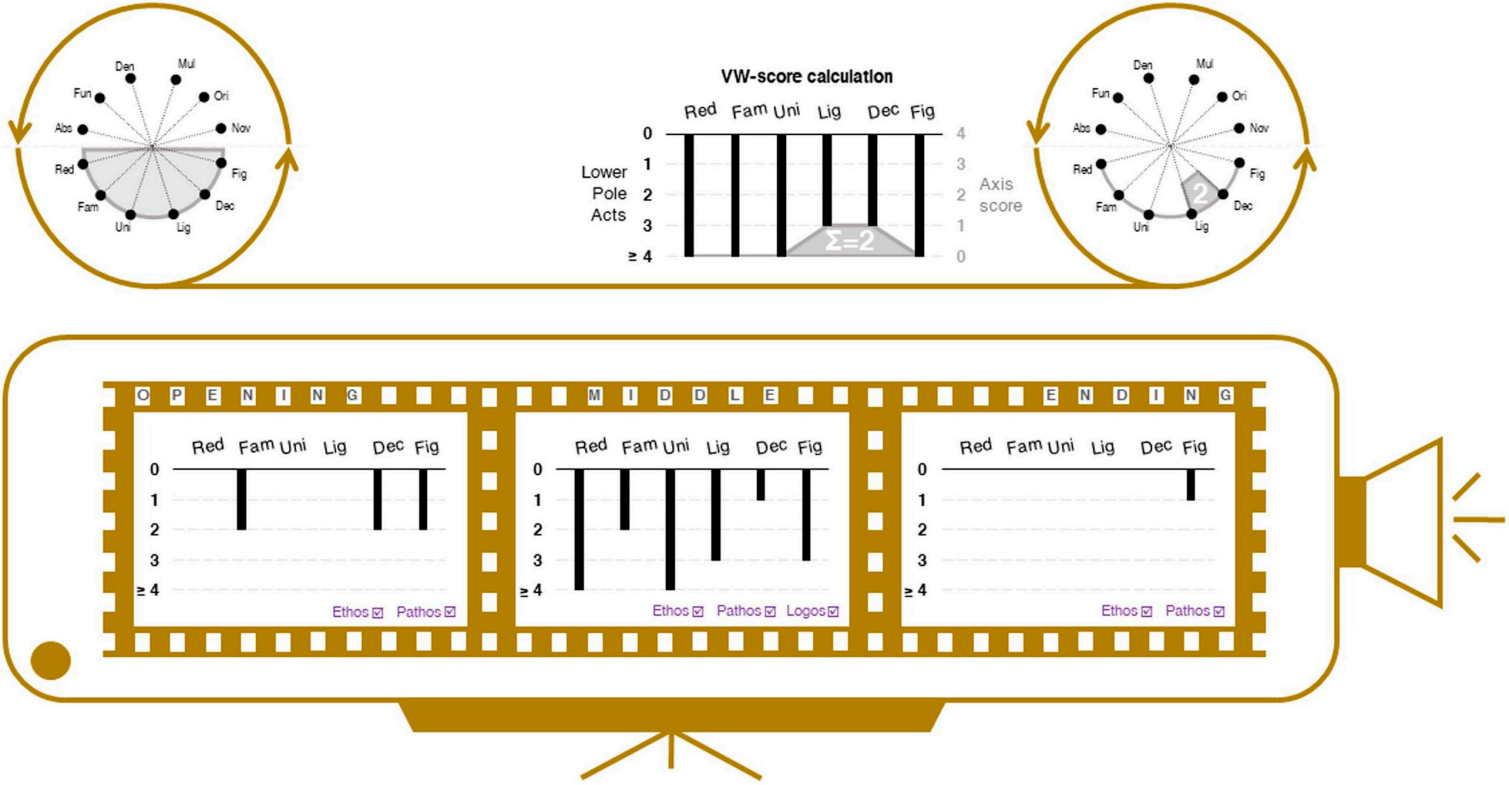
The visual storytelling framework and visual storytelling of vaccine safety, by Botsis et al. 2020: An application of the visual storytelling framework for the construction of a visual story on vaccine safety. Reprinted from [44], Copyright (2023), with permission from Elsevier.

In recent years, visualization researchers have begun to explore communication for medical knowledge dissemination. They have aimed at applying visualization for narrative immersion in knowledge dissemination and the engagement of patients or the public. Botsis et al. [44] proposed a theoretical framework for visual storytelling in biomedical science. The framework included the 4 main phases of visual story development: target audience identification, audience health literacy evaluation, visual story design, and visual story production; see Fig. 9. Hence, it supports designers in making specific design decisions to map audiences’ high or low health literacy, making knowledge dissemination more understandable based on individual differences. Two visual stories on vaccine safety and cancer immunotherapy were presented and designed according to the proposed framework and phases.

Madathil and Greenstein [45] explored approaches to designing comprehensible healthcare public reports. The work developed a presentation format to enhance consumers’ engagement with public reports and support their informed healthcare decisions. Following their experimental exploration, it was demonstrated that presenting a narrative within the context of a consumer’s personal experiences can engage the public more in data employment and subsequent decision-making processes. Meanwhile, portraying the care quality metrics test at every step of a decision-making task before the final choice supports the use of public report information. However, comprehensive guidelines for narrative presentation should be explored in future work.

**Fig. 10.**
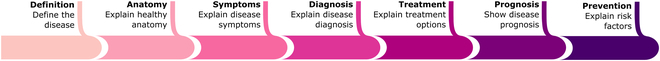
The 7-stage template for narrative medical visualization of disease data by Garrison et al. 2022. Reprinted from [48], Copyright (2023), with permission from Elsevier.

Similarly, Meuschke et al. [48] focused on the general public (nonexperts) and demonstrated the potential of narrative medical visualization to engage the same. They illustrated a 7-stage template as an essential structure for data-driven narrative medical visualization, as shown in Fig. 10. Three interactive web-based medical stories for 3 diseases were designed and evaluated regarding understanding, remembering stories, data credibility, user engagement, and motivation.

#### Visually connecting people with diseases

Junior et al. [46] paid attention to fostering people’s empathy and connecting people emotionally with diseases. They presented a storytelling empathy game to visualize breast cancer journeys. A 3D game was designed based on patients’ real-life experiences. Two characters, a female cancer patient and a male oncologist, formed the center point of the game story. Based on patients’ stories, the game focused on building up breast cancer issues. The game playtesting was used to evaluate the gaming experience. The questionnaire with closed and open questions was implemented to evaluate empathy evoking and emotional connection. For example, play testers’ own feeling and emotions during gameplay and their perceived emotions in the patient character were collected during the questionnaire. The 2 groups of collected emotions were compared. Questions about engagement and immersion in the game were also included. The results showed that storytelling games could share patients’ scenarios to help patients, their families, friends, and health professionals understand patients’ priorities and preferences, thereby delivering genuine patient-centric care. However, it has been learned that evaluating empathy within a gameplay is subject and difficult. Moreover, more playtesting data and interaction options are necessary for further evaluation.

### Visualization for medical conversation training in education

**Fig. 11.**
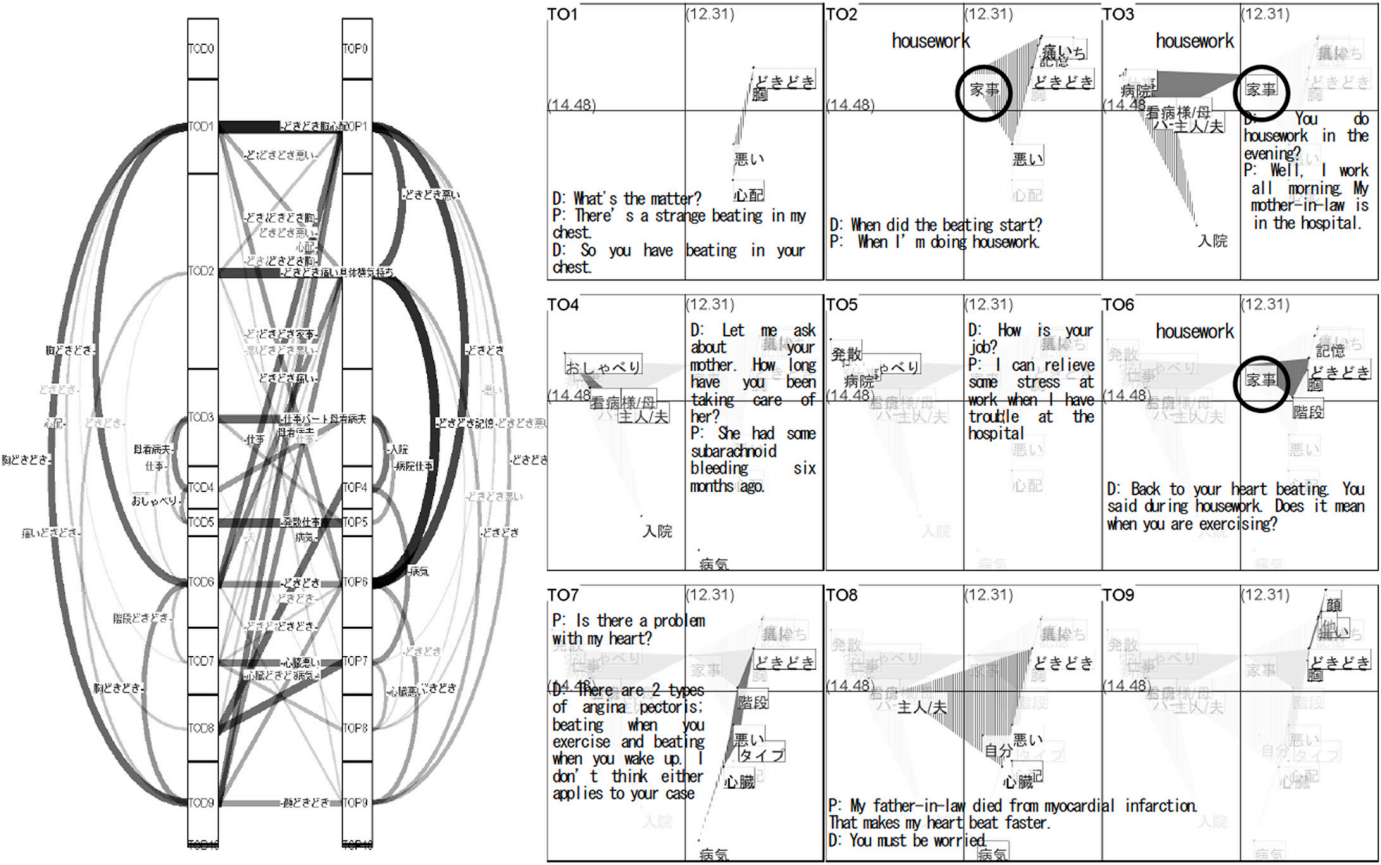
Topic structure and concept slices by Koyama et al. Left: A topic structure, where the thickness of the lines between topics indicates the similarities of topics. Right: A concept space, in which TO1 to TO9 are the concept slices in the dialogue and where different topic areas are displayed in the slices. © [2023] IEEE. Reprinted, with permission, from [28].

In medical education, close reading and creative writing improve students’ communication abilities and empathy with patients [9]. Rather than using these 2 typical genres of methods, Babaian [35] utilized drawing and comic books to illustrate visually animated storytelling for a surgical procedure. He believed that visual storytelling could help introduce the important role of empathy, deepen medical students’ relationships, and strengthen their passion for the biological storytelling subject. Similarly, Koyama et al. [28] attempted to support the medical education of doctor-patient dialogues. Their visualization method provided doctors with a semantical topic structure of the dialogue, as shown in Fig. 11. Meanwhile, this topic structure supported the presentation of context and flow of dialogues. Subsequently, insights could be extracted from the stories that stemmed from these dialogues. This method proved beneficial for applying to medical education to develop students’ abilities in patient–physician conversation. However, future work needs to improve this method to increase its readability.

### Visualization for psychotherapy and emotional wellness enhancement

Regarding mental health, narrative and storytelling have been shown to encourage recovery and rehabilitation [53–56]. Therefore, visualization methodologies were applied in this domain to support these interventions, including pictures and images in narrative therapy [57,58]. In our review study, however, we exclude the type of therapy based on pictures and images since what we focus on is the exploration of visualization for data, information, and narratives.

Coyle et al. [27] studied adolescent engagement requirements during psychotherapy. They defined a computer-aided communication model in psychotherapy and designed an online 3D computer game to increase engagement and empower adolescents’ self-control of their therapy. Meanwhile, the game helped adolescents construct and share their own stories and see their peers’ narratives and stories. They designed the game by choosing detective stories and metaphors and mapping the therapeutic conversational strategies of the solution-focused therapy model. Hence, adolescents could set their own therapeutic goals and achieve therapy by following the structured storytelling in the game.

Choudhury et al. [33] aimed to help the public visualize their self-narrative or “behavioural fingerprints” on social media. They believed that this kind of emotional reflection by individuals, based on their linguistic usage and social activities online, could be an unobtrusive mechanism to enhance emotional wellness. The visualization utilized an analogy of the moon’s phases to represent the positive and negative effects of trends in people’s Twitter posts. Meanwhile, they presented people’s linguistic style usage over all their posts and the relative percentages of each style. The work intends to reveal the trends of emotions and behavior to help people manage their feelings effectively and cope healthily with any adverse effects.

## Solutions of Visualization Applied in Narrative Medicine

As can be seen from Table 2, the visualization solutions of “Pictograms/graphs” and “Interface/dashboard/web pages” were the most frequently employed, with a total of 13 and 12 studies, respectively. “Timeline” was utilized by 6 studies. Both “Games” and “3D Modelling” were applied in 3 studies. The other forms, e.g., “Digital storytelling”, “Web APP”, and “Comic books”, were separately employed in one work.

**Table 2.** Forms and solutions of visualization by domains. The study labels shown in italic and bold represent the works in the most recent 5 years (after 2018).

Forms and solutions of visualization	Domains				Total
	Narrative text visualization in EHR	Communication	Therapy or intervention	Medical education	
S1: Pictogram / graph	(7) ***(1) (2) (3)***	(13)(14) ***(10) (12) (15) (16) (18)***	(21)	(20)	13
S2: Interface / dashboard / web pages	(4)(6)(7)(8) ***(2)***	(13) ***(10) (12) (15) (16) (18)***	(21)		12
S3: Timeline	(2)(7)(4)(5)(9)	***(12)***			6
S4: Game		***(11**)**(17)***	(22)		3
S5: 3D Modeling		***(15)(17)***	(22)		3
S6: Digital storytelling		***(11)***			1
S7: Web APP		***(11)***			1
S8: Comic book / graph novel				(19)	1

It is worth mentioning that 12 studies (2, 4, 7, 10, 11, 12, 13, 15, 16, 17, 18, and 21) utilized more than one form of visualization. For example, 4 (2, 7, 12, and 15) simultaneously included pictograms, graphs, and timelines or 3D modeling in their interface panels. In addition, Study 11 [40] explored visualization by encompassing digital storytelling, a game, and a web app in one solution. The following section will discuss the leading solutions of visualization applied in the narrative medicine practice and the implementation trends.

### Multiple views and structures of interface/dashboard/webpages (S2)

Interfaces, dashboards, or webpages were the primary solution for visualizing narratives in EHR and communication. The 5 studies (2, 4, 6, 7, and 8) for narratives in EHR organized and presented an overview, history, and new information of clinical notes into different panels and blocks in their interfaces and supported multilevel granularity of information by variable interaction. They generally comprised 3 or 4 tiled panels on one screen, which included a panel for demographics or profile information and a control or settings tools panel for keywords or display level selection. Moreover, the text or document panel was always the main panel for presenting new or historical information. Colors were frequently used to highlight the relative texts (through the background color of texts) and to distinguish different semantic types by specific color codes.

In the communication domain, studies (10, 12, 13, 15, 16, and 18) with dashboards or webpages focused much more on the structure or sequence of the pages (views). These works featured several views (pages) and presented these views in a specific order, where the order reflected the structure of the storytelling and narrative visualization. For example, Study 18 [44] proposed a theoretical framework with 4 primary phases for visual storytelling in biomedical science. Study 10 [43] constructed the 3 aspects of information (bio, psycho, and social) with the martini glass structure of narrative visualization [26]. The structure was designed to begin with “author-driven” storytelling to tutor users implicitly through a linear sequence and then continue with “reader-driven” storytelling to give users more freedom to explore and interact. In line with the martini glass structure, the dashboard presented the 3-story slices with bubble charts, pictograms of the human body and emoticons, radar charts, and choropleth maps. Study 12 [41] separately explored 2 dashboard structures (through a timeline and tabular layout) for daily reports and situation reviews. More recently, Study 15 [48] proposed a 7-stage template as an essential structure for narrative medical visualization.

### Pictograms/graph for information readability (S1)

Most of the studies (2, 7, 10, 12, 13, 15, 16, 18, and 21) featuring dashboards or webpages utilized a variety of graphs or charts (e.g., radar, pie, bar charts, choropleth maps, and road maps) since they supported highlighting, comparing critical information and representing processes of treatment or decision-making. Moreover, a pictogram is a readable visual form that can enhance information understanding. For example, Study 14 [37] employed an array of pictograms as its basic visualization form for improving Bayesian reasoning. Meanwhile, the same study explored the practical solutions for presenting expression with pictograms, e.g., Vis-Only, Control+Vis, and Storyboarding. Similarly, Study 21 [33] intuitively presented people’s emotional expression through pictograms featuring moon phases.

Study 20 [27] proposed a specific graph to visualize the structure of dialogue topics, featuring 2 vertical lists of divided topics from doctor to patient, see Fig. 11. The system employed lines with different thicknesses between topics to indicate their similarities. The graph illustrated the topic relationship during the patient–physician dialogues and highlighted the narrative medicine practice process and images of patients’ experiences. However, the weak readability of the graph blocked understanding of the topic structure. An improved visualization method should be used in future work.

### Specific timeline styles for historical and causal information (S3)

In the studies (2,4,7, and 12), which provided their solutions in dashboards or webpages, a timeline was commonly used for history information display, which could integrate pictograms, shapes, sizes, and colors to present the retrieved text information and provide an overview of historical narrative records. Meanwhile, interaction in timelines, such as scrolling, mouse-over, selecting, and zooming, can support multilevel granularity of information exploration.

Specific styles of timelines were explored. For example, Study 5 [34] developed V-model to visualize narrative patient history in EHR chronologically; see Fig. 4. The timeline organized a V-shaped structures temporally. Every single V-shaped structure conveyed the situation of a clinical event. Thus, it could present a problem–action relationship and was able to show the causal events for temporal reasoning. Meanwhile, the timeline was dynamic and scalable and could display uneven granularity (hour or minute levels) expressions in one timeline. As a result, the V-model was especially effective in reading long histories and addressing the issue of nonexplicit temporal expression (nonexplicitness). However, it took more time to recognize qualitative temporal relations than conventional timeline displays. So, improving the simplicity of the V-model would be challenging work.

### Integration of digital storytelling and web APP (S6 and S7)

In visualization, storytelling technology presents a visual data story and dynamic relationships between story nodes (pieces) by interaction [59,60]. Digital storytelling is a visualization solution that uses multimedia tools (graphics, audio, video, and animation) to tell stories more engagingly [40]. Study 11 [40] integrated digital storytelling with game-based approaches to improve the motivation and engagement of young patients. The components of animations with different goals, scenarios, and characters were defined and inserted into the system. Based on these animation components, young patients could create their own stories by selecting their personal goals, scenarios, leading actors, and characters in the defined structures. Meanwhile, a web app was developed as a content management system. Hence, the multiple stakeholders, including young patients, caregivers, parents and families, could read the patients’ individual stories to monitor and discover their activities and emotional states.

### 3D serious game (S4 and S5)

Game and 3D modeling technologies were recently explored in narrative medicine practice. Study 11 [40] integrated the serious game approach into digital storytelling, which is simulation games not for entertainment and enjoyment. Hence, the games simulated professional training or informal learning. Study 17 [46] designed a 3D game to foster people’s empathy for disease. Furthermore, Study 22 [27] combined theories of play therapy, therapeutic storytelling, and solution-focused therapy (a goal-oriented, strengths-based model of psychotherapy). They designed an online 3D computer game. In the game strategies, the trainees talk to 5 master detectives in 5 game areas and accomplish tasks to collect keys. A detective notebook was provided to guide the trainees in setting the overall therapeutic goal of the game. Meanwhile, the same notebook could be helpful in future therapy sessions.

## Future Opportunities

**Fig. 12.**
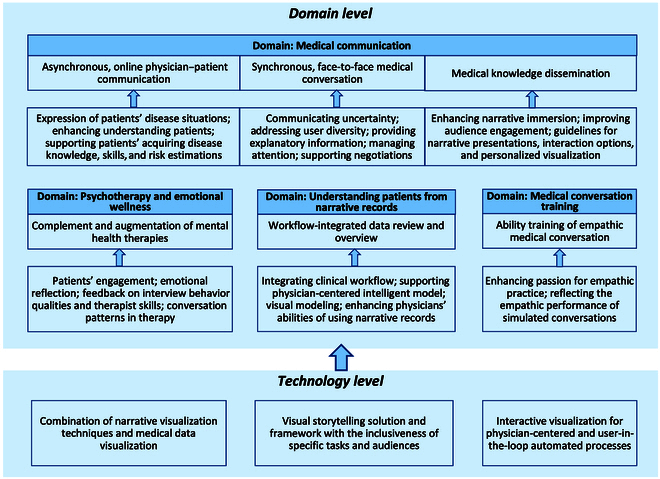
Future work framework of visualization in narrative medicine.

We constructed a framework for conceptualizing the future work of visualization in narrative medicine from 2 levels: domain and technology. Firstly, it focuses on 4 visualization application domains of medical communication, psychotherapy and emotional wellness, understanding patients from narrative records, and medical conversation training. The 4 domains belong to the 2 main directions of narrative medicine [5–7]: narrative competence training of physicians and other medical workers and students (the domain of medical conversation training) and narrative-based medical practice (the domains of medical communication, psychotherapy and emotional wellness, and understanding patients from narrative records). Secondly, the relevant visualization studies extract the main future topics about technology developments and applications. See Fig. 12.

### Domain level

#### Psychotherapy and emotional wellness

Narrative and storytelling are the traditional methodologies employed in psychotherapy in mental health and emotional wellness. Therefore, according to Table 1, it is evident that the earliest study, Study 22 [27], belonged to the domain of psychotherapy enhancement, which focused on the problem of adolescents’ engagement with mental healthcare. Study 22 [27] and Study 21 [33] explored visualization tools to support emotional reflection and psychological health.

In recent years, some work has started to explore visualization to support and enhance the process of mental health therapy conversations. For example, an interactive visualization [61] was designed to integrate machine learning to provide psychotherapists with summary feedback reports on motivational interviewing quality measurements for one-on-one therapy. The reports included the motivational interviewing fidelity score, adherence score, and specific behavior qualities. The utility of the report system should be explored in the future. For group therapy, Thomas [62] designed prototypes for a visualization tool to support therapists with group information, session dynamics, transcript inspection, and therapist skills. Integrating the changing of the 4 elements with time will be worthy of exploration to reveal essential patterns in therapies.

#### Medical communication

As outlined in Table 1. Since 2016, 9 studies have been conducted in the domain of communication. It indicates a trend of additional focus and interest in visualization for communication in narrative medicine. Within the studies for physician–patient communication, Studies 10 and 11 [40,43] focused on the context of asynchronous and online communication. Studies 13 and 14 [37,38] explored improving patients’ understanding of physician–patient risk communication, which is also asynchronous. Finally, Study 12 [41] proposed visualization tools for recording and reviewing patients’ narratives in daily living. These studies indicated that further visualization research should concern expressing patients’ disease situations (biological states and psychological and emotional states), enhancing physicians’ adequate understanding of patients, and supporting patients’ acquiring disease knowledge and skills and the uncertainty of risk estimations.

A further opportunity for future visualization research could be exploring face-to-face synchronous medical conversations. Study 12 [41] began to be concerned with face-to-face scenarios, as in synchronous medical conversations. They visualized patient-generated data to support the context before and between 2 medical encounters. Nevertheless, the face-to-face scenario was not their central focus. Few studies of visualization focused on on-site face-to-face medical conversations. Recently, we completed an empirical study on this matter [63]. As a result, it was demonstrated that patients and physicians accepted and had specific requirements for visualizing on-site synchronous encounters. Subsequently, 5 opportunities for future studies were suggested, including communicating uncertainty, addressing user diversity, providing explanatory information, managing attention, and supporting negotiations [63].

Visualization for dissemination is another potential trend in medical communication. As shown in Table 1, there have been 4 studies since 2020. More research should be conducted to enhance narrative immersion in medical knowledge dissemination and improve audience engagement. Comprehensive guidelines for narrative presentation, more interaction options, and more personalized visualization were suggested for future research agendas [45,46,48,64]. Storytelling technology supports conveying medical information to the general public. The characters and protagonists of the story and the patient-centered story structure were worth further exploring in the future [65].

#### Understanding patients from narrative records

To understand patients by narrative records visualization, exploring strategies for integrating visualization into existing clinical workflows is critical in future work [42]. Moreover, interactive visualization could support a more physician-centered NLP model. For example, this could include user-in-the-loop iterative refinement of an automated process. Such visualization could be helpful for automation transparency and verification [42]. Furthermore, visual modeling of unstructured clinical narrative texts is an intuitive method for physicians to structure clinical notes, present relative information, and help using narrative text [47]. Hence, when integrated with NLP or other intelligent technologies, interactive visualization, which enhances physicians’ ability to use EHR, is worth exploring in the future.

#### Medical conversation training

Some studies have explored visualizing empathic medical conversations for medical students’ education. The research topics could include those following simulated medical encounters, enhancing students’ passion for empathic practice [35], reflecting semantical topic structure [28], conversation habits [66], individual contributions [67], and empathy levels [68]. Increasing the readability of the visualizations is always crucial for future work. Moreover, improved outcomes of narrative medicine training, for example, narrative competence, relationship building, reflection and empathy capabilities, and so on, are worth considering in future visualization exploration to support the evaluation of narrative medicine training programs [69].

### Technology level

Concerning the visualization of the combination of narrative techniques and medical data, Meuschke et al. [48] suggested a research agenda for the future. For example, further exploration of more templates should be derived from medical concepts and healthcare models [43]. Further evaluation of design decisions should be continued, e.g., strategies, genres and narrative patterns. They mentioned that in a doctor–patient conversation, the patient could be informed about his/her condition by more personalized visualization to help understand diagnostic and therapeutic procedures.

Visual storytelling solutions combine multiple components with photographs, graphics, animation, video, text, and other mediums [44]. It presents specific information through multisection visual representations [70]. Hence, the technologies of visual story architecture, visualization components selection, and story building require consideration of the characteristics of target audients (health literacy, numeracy, and psychosocial parameters) and their different purposes and tasks [38,43,44,71]. Focusing on specific clinical tasks, for example, risk communications, medical dissemination, or others, visual storytelling solutions and frameworks are required for further exploration.

User-centered Interactive visualization tools assisted physicians in reviewing and overview narratives in EHR. In recent years, physician-centered NLP models and user-in-the-loop automated processes have attracted more explorations. Hence, visual modeling of clinical narratives [47] and user-in-the-loop iterative refinement of an automated process [42] are the future targets in supporting physicians using EHR. Moreover, when supporting physician–patient communication, So et al. [43] concluded that visual communication with human-in-the-loop was a future exploration. For medical storytelling, an artificial intelligence (AI)-assisted iterative pipeline for data-driven medical story design, for example, character design of the story and others, is a valuable future direction with the development of generative AI technology [72].

## Conclusion

In this work, to explore and define the visualization research domains for narrative medicine, 22 visualization studies were collected and analyzed. We identified 4 domains and contexts in which visualization has been explored for narrative medicine, including understanding patients from narrative records, medical communication, medical conversation training in education, and psychotherapy and emotional wellness enhancement. From the analysis, it is evident that visualization can support the practice of narrative medicine in light of physicians’ requirements and workflows. In addition, except for the general solutions applied in the studies featured dashboards, webpages, timelines, graphs, and charts, integrating the sequence of dashboard pages with the storytelling structure and combining the serious game approach into digital storytelling are valuably explored in the future. Meanwhile, for both perspectives of visualization technology and application domains in narrative medicine, several potential research opportunities are highlighted and worthy of further exploration. In the future, more research should be done regarding these aspects to support narrative medicine practice.
